# Which low urgent triaged febrile children are suitable for a fast track? An observational European study

**DOI:** 10.1136/emermed-2023-213375

**Published:** 2024-01-18

**Authors:** Chantal D Tan, Clementien L Vermont, Joany M Zachariasse, Ulrich von Both, Enitan D Carrol, Irini Eleftheriou, Marieke Emonts, Michiel van der Flier, Jethro Herberg, Benno Kohlmaier, Michael Levin, Emma Lim, Ian K Maconochie, Federico Martinon-Torres, Ruud G Nijman, Marko Pokorn, Irene Rivero-Calle, Aleksandra Rudzāte, Maria Tsolia, Werner Zenz, Dace Zavadska, Henriette A Moll

**Affiliations:** 1 General Paediatrics, Erasmus MC Sophia Children's Hospital, Rotterdam, The Netherlands; 2 Section of Paediatric Infectious Diseases and Immunology, Erasmus MC Sophia Children's Hospital, Rotterdam, The Netherlands; 3 Paediatric Infectious Diseases, University Children's Hospital at Dr. von Haunersches Kinderspital, LMU Munich, Munich, Germany; 4 Section of Paediatric Infectious Diseases and Immunology, Alder Hey Children's NHS Foundation Trust, Liverpool, UK; 5 Department of Clinical Infection, Microbiology and Immunology, University of Liverpool Institute of Infection Veterinary and Ecological Sciences, Liverpool, UK; 6 Paediatrics, P and A Kyriakou Children’s Hospital, Athens, Greece; 7 Paediatric Immunology, Infectious Diseases & Allergy, Great North Children's Hospital, Newcastle upon Tyne, UK; 8 Newcastle University Translational and Clinical Research Institute, Newcastle upon Tyne, UK; 9 Paediatric Infectious Diseases and Immunology, Wilhelmina Children's Hospital University Medical Centre, Utrecht, The Netherlands; 10 Paediatric Infectious Diseases and Immunology, Amalia Children's Hospital, Nijmegen, The Netherlands; 11 Section of Paediatric Infectious Diseases, Imperial College London, London, UK; 12 Department of General Paediatrics, Medical University of Graz, Graz, Austria; 13 Imperial College London, London, UK; 14 Paediatric Emergency Medicine, Imperial College Healthcare NHS Trust, London, UK; 15 Genetics, Vaccines, Infections and Paediatrics Research group, Hospital de Clinico Universitario de Santiago de Compostela, Santiago de Compostela, Spain; 16 Department of Infectious Diseases, University Medical Centre Ljubljana, Ljubljana, Slovenia; 17 Paediatrics, Children's Clinical University Hospital, Riga, Latvia; 18 Erasmus MC Sophia Children's Hospital, Rotterdam, The Netherlands

**Keywords:** triage, emergency department, pediatrics

## Abstract

**Background:**

The number of paediatric patients visiting the ED with non-urgent problems is increasing, leading to poor patient flow and ED crowding. Fast track aims to improve the efficiency of evaluation and discharge of low acuity patients. We aimed to identify which febrile children are suitable for a fast track based on presenting symptoms and management.

**Methods:**

This study is part of the Management and Outcome of Fever in children in Europe study, which is an observational study including routine data of febrile children <18 years attending 12 European EDs. We included febrile, low urgent children (those assigned a triage acuity of either ‘standard’ or ‘non-urgent’ using the Manchester Triage System) and defined children as suitable for fast track when they have minimal resource use and are discharged home. Presenting symptoms consisted of neurological (n=237), respiratory (n=8476), gastrointestinal (n=1953) and others (n=3473, reference group). Multivariable logistic regression analyses regarding presenting symptoms and management (laboratory blood testing, imaging and admission) were performed with adjustment for covariates: patient characteristics, referral status, previous medical care, previous antibiotic use, visiting hours and ED setting.

**Results:**

We included 14 139 children with a median age of 2.7 years (IQR 1.3–5.2). The majority had respiratory symptoms (60%), viral infections (50%) and consisted of self-referrals (69%). The neurological group received imaging more often (adjusted OR (aOR) 1.8, 95% CI 1.1 to 2.9) and were admitted more frequently (aOR 1.9, 95% CI 1.4 to 2.7). The respiratory group had fewer laboratory blood tests performed (aOR 0.6, 95% CI 0.5 to 0.7), were less frequently admitted (aOR 0.6, 95% CI 0.5 to 0.7), but received imaging more often (aOR 1.8, 95% CI 1.6 to 2.0). Lastly, the gastrointestinal group had more laboratory blood tests performed (aOR 1.2. 95% CI 1.1 to 1.4) and were admitted more frequently (aOR 1.4, 95% CI 1.2 to 1.6).

**Conclusion:**

We determined that febrile children triaged as low urgent with respiratory symptoms were most suitable for a fast track. This study provides evidence for which children could be triaged to a fast track, potentially improving overall patient flow at the ED.

**WHAT IS ALREADY KNOWN ON THIS TOPIC**

- Poor patient flow and crowding are major issues at the ED.
- A fast track intervention for patients with non-urgent problems improves patient flow at the ED and is a promising intervention to reduce length of stay and has been found to increase patient satisfaction.

**WHAT THIS STUDY ADDS**

- In a multicentre observational study in Europe, we determined that among febrile children triaged as low urgent, those with respiratory symptoms were most suitable for a fast track.

**HOW THIS STUDY MIGHT AFFECT RESEARCH, PRACTICE OR POLICY**

- This study provides evidence for which children could be triaged to a fast track, potentially improving overall patient flow at the ED.

## Introduction

The number of paediatric attendances to the ED with non-urgent problems is increasing in Europe, leading to poor patient flow and crowding.^1 2^ Non-urgent patients visiting the ED leads to more resource use, higher medical costs and higher work pressure for healthcare workers. A fast track intervention to improve patient flow at the ED is a promising intervention to reduce length of stay at the ED and has been found to increase patient satisfaction.^3–9^

A fast track is a separate healthcare pathway for the assessment and treatment of patients who need a lower level of care in a dedicated area near the ED,^4 10 11^ allowing more effective management of patients with non-urgent problems.^12^ In order to implement a fast track for non-urgent patients, an absolute requirement is having a reliable triage system. Triage at the ED is used to prioritise patients based on their clinical urgency and to ensure that patients are seen in order of clinical priority rather than in order of attendance.^13^ It can therefore be used to identify patients with less urgent problems who can safely wait longer until doctors’ assessment at the ED or who can be seen by another caregiver such as a general practitioner or nurse (practitioner).

The Manchester Triage System (MTS) is the most commonly used triage system in Europe; it categorises patients into one of five triage categories based on presenting symptoms.^14^ According to a prospective observational study in two paediatric emergency care settings, MTS can safely identify less urgent patients. Fever is one of the most common presenting symptoms in children visiting the ED, accounting for 20% of all paediatric ED visits.^15^ Therefore, implementing a fast track for febrile children may have large impact on patient flow by shortening the length of stay and waiting time at the ED.

The aim of our study is to determine which low urgent febrile children triaged by MTS as low urgency are suitable for assessment in a fast track. This approach is based on objective classification by the MTS and differs from forms of streaming where patients are directed to a healthcare provider after brief clinical assessment or telephone contact.^16^ Identifying febrile children suitable for a fast track may allow them to be treated in a lower resource setting, shortening their stay and potentially improving patient flow in the rest of the ED.

## Methods

### Study design

This is a secondary analysis of the Management and Outcome of Fever in children in Europe (MOFICHE) study, which is embedded in the Personalised Risk assessment in Febrile illness to Optimise Real-life Management across the European Union project.^17 18^ The MOFICHE study is an observational multicentre study assessing management and outcome of febrile children using routinely collected data of 12 EDs in 8 European countries (Austria, Germany, Greece, Latvia, the Netherlands n=3, Slovenia, Spain, the UK n=3). The hospital characteristics are described in previous studies.^18 19^

### Study population and setting

Children up to 18 years with fever (temperature ≥38℃) measured at the ED or a history of fever within 3 days before the ED visit were included in the MOFICHE study. For this secondary analysis, we included the nine EDs who use the MTS for allocating triage urgency levels to patients, namely EDs in Austria, Germany, Latvia, the Netherlands (n=2), Spain, and the UK (n=3). Subsequently, from these EDs we included children who were triaged as low urgent (those assigned a triage acuity of either ‘standard’ or ‘non-urgent’ using the MTS) since we hypothesise that a proportion of these children are suitable for assessment in a fast track. Children with known comorbidities and with missing data on disposition were excluded.

### Patient and public involvement

Patients and public were not involved in the design and conduct of the study.

### Triage urgency level

The MTS consists of 52 flow charts based on the patients’ presenting problem such as abdominal pain.^13 14^ The most appropriate flow chart is chosen by triage nurses to prioritise patients on clinical urgency. Each flow chart consists of specific discriminators and categorises patients in one of the five triage categories, which are linked to a maximum waiting time for doctors’ assessment. The five MTS urgency categories are: immediate (maximum waiting time 0 min), very urgent (maximum waiting time 10 min), urgent (maximum waiting time 60 min), standard (maximum waiting time 120 min), non-urgent (maximum waiting time 240 min). For this study, we used predefined three-category triage levels consisting of the categories ‘high urgent’, ‘intermediate urgent’ and ‘low urgent’.^20^ The MTS categories very urgent and immediate were classified as high urgent, urgent was classified as intermediate urgent and patients allocated to standard or non-urgent were classified as low urgent.

### Data collection

Data were routinely collected from electronic health records for at least 1 year during the MOFICHE study period from January 2017 to April 2018. Period of active data collection per month differed in the participating hospitals ranging from 1 week per month to the entire month. Characteristics of the participating hospitals are shown in online supplemental appendix A. Data collected included patient characteristics (age, gender, presenting symptoms, comorbidity (chronic condition expected to last at least 1 year^21^), referral status, triage urgency, visiting hours, previous medical care, previous antibiotic use, vital signs (HR, RR, oxygen saturation, temperature), diagnostic tests performed in the ED (laboratory blood testing, imaging), antibiotic prescription (at the ED or first day of admission) and disposition. Presenting symptoms were categorised into four groups: neurological (febrile convulsions, meningeal signs or focal neurological signs), respiratory (runny nose, sore throat or coughing), gastrointestinal (diarrhoea or vomiting) and other (eg, rash, urogenital symptoms) presenting symptoms. Referral status was dichotomised into self-referred and referred (referral by general practitioner or other hospital or emergency medical services). Previous medical care was defined as a visit to a healthcare setting (general practitioner or ED) in the previous 5 days, and previous antibiotic use was defined as therapeutic antibiotic use in the last 7 days. Visiting hours were categorised as office hours and out-of-office hours, with out-of-office hours defined as ED attendances in weekends or between 17:00 hours and 08:00 hours on weekdays. Tachypnoea and tachycardia were defined according to age-specific cut-off values as described in APLS guidelines.^22^ The focus of infection and cause of infection were retrospectively assigned by the local research teams. The focus of infection was categorised into respiratory tract, gastrointestinal tract, urinary tract, childhood exanthema/flu-like illness, soft tissue/skin/musculoskeletal, sepsis/meningitis and other (eg, undifferentiated fever). The cause of infection was determined using a previously published phenotyping algorithm, which combines clinical symptoms and diagnostic results.^18^ Patients were categorised as presumed bacterial, presumed viral, unknown bacterial/viral or other (eg, inflammatory illness). Children with a mixed bacterial and viral infection were classified as bacterial (online supplemental appendix B).

10.1136/emermed-2023-213375.supp1 Supplementary data

### Outcome measures

We defined children suitable for a fast track when resource use at the ED is minimal and when there is no need for admission. This definition was based on previous literature and on expert opinions of the research group including paediatricians.^5 11^ Resource use included laboratory blood testing and imaging performed at the ED. Laboratory blood tests included markers of infection; C reactive protein (CRP), procalcitonin and white blood cell (WBC) count. Imaging included X-ray, ultrasound, MRI scan and CT scan. We defined children with laboratory blood testing, any kind of imaging or being admitted as not suitable for a fast track.

### Data analysis

Descriptive statistics were used for patient characteristics and management. We performed univariable and multivariable logistic regression analyses for the association between presenting symptoms and laboratory blood testing, imaging and admission. We adjusted the analyses for the confounders of age, sex, referral status, previous medical care, previous antibiotic use, visiting hours and ED setting. Additionally, we stratified the analysis for ED settings with low (22%–57%) and high (65%–89%) prevalence of low urgent triaged children during the study period. Subgroup analysis describing frequency of patient management stratified by age groups were performed when relevant for a fast track. We used multiple imputation with the MICE package in R for missing data on clinical covariates. Data were analysed using SPSS software V.25.0 and a p value <0.05 was considered statistically significant.

## Results

### Patient population and characteristics

A total of 29 588 febrile children attended the 9 European EDs, of which 16 683 (56%) were triaged as low urgent. The proportion of low urgent triaged children ranged from 22% to 73% across the EDs, and the three triage urgency categories per ED setting are shown in online supplemental appendix C. After excluding children with comorbidity (14%) and missing data on disposition (0.1%), the population for analyses consisted of 14 139 children. Table 1 describes the patient characteristics of the study population with a median age of 2.7 years (IQR 1.3–5.2) and 54% being boys. Most of the ED attendances were during out-of-office hours (70%) and the majority consisted of self-referrals (68%). Respiratory symptoms were the most common presenting symptom (60%), whereas neurological symptoms were least common (2%). Abnormal vital signs varied from 0.6% to 14% and the median duration of fever was 1.5 days. Patient characteristics per presenting symptom group are shown in online supplemental appendix D. The percentage of self-referrals was the lowest in the neurological group and all other characteristics were comparable between the presenting symptom groups.

**Table 1:** Patient characteristics (n=14 139)

	Low triaged febrile children N=14 139	Missing (%)
Age* (years)	2.7 (1.3–5.2)	
Gender (boys)	7613 (54)	
Visit hours (out of office)	9852 (70)	
Referral (self-referred)	9630 (68)	556 (4)
Previous medical care	3381 (24)	671 (5)
Previous antibiotic treatment	1486 (11)	294 (2)
Presenting symptoms		
Neurological	237 (2)†	
Respiratory	8476 (60)	
Gastrointestinal	1953 (14)	
Other	3473 (25)	
Ill appearance	1653 (12)	514 (4)
Vital signs		
Tachycardia	2019 (14)	1317 (9)
Tachypnoea	1346 (10)	2465 (17)
Hypoxia	78 (0.6)	2788 (20)
Duration of fever (days)*	1.5 (0.5–3)	1067 (8)

Absolute numbers and percentages (%) are given.

*Median and (IQR 25–75).

†87% status after febrile convulsion.

### Management and diagnosis

Table 2 depicts the management, focus of infection and the presumed cause of infection of our study population. Laboratory blood tests were performed in 34% of the visits, of which CRP and WBC count were most frequently performed (33%). Thirteen per cent received any kind of imaging, 13% were admitted and 31% received antibiotic treatment. The majority had a respiratory focus of infection (69%) and a presumed viral infection (50%).

**Table 2:** Management and working diagnosis

	Low triaged febrile children N=14 139
Laboratory blood tests	4740 (34)
CRP	4659 (33)
PCT	252 (2)
WBC	4665 (33)
Imaging	1864 (13)
X-ray	1603 (11)
Ultrasound	338 (2)
CT scan	40 (0.3)
MRI scan	18 (0.1)
Admission	1840 (13)
Left without being seen	70 (0.5)
Antibiotic treatment	4395 (31)
Focus of infection	
Respiratory	9702 (69)
Gastrointestinal	1317 (9)
Urinary	374 (3)
Childhood exanthema/flu-like illness	746 (5)
Soft tissue/Skin/Musculoskeletal	382 (3)
Sepsis/Meningitis	21 (0.1)
Other	1596 (11)
Cause of infection	
Presumed bacterial	3375 (24)
Unknown bacterial/viral	2348 (17)
Presumed viral	7034 (50)
Other	1194 (8)

Absolute numbers and percentages (%) are given.

CRP, C reactive protein; PCT, procalcitonin; WBC, white blood cell.

### Association between presenting symptoms and management

**Figure 1:**
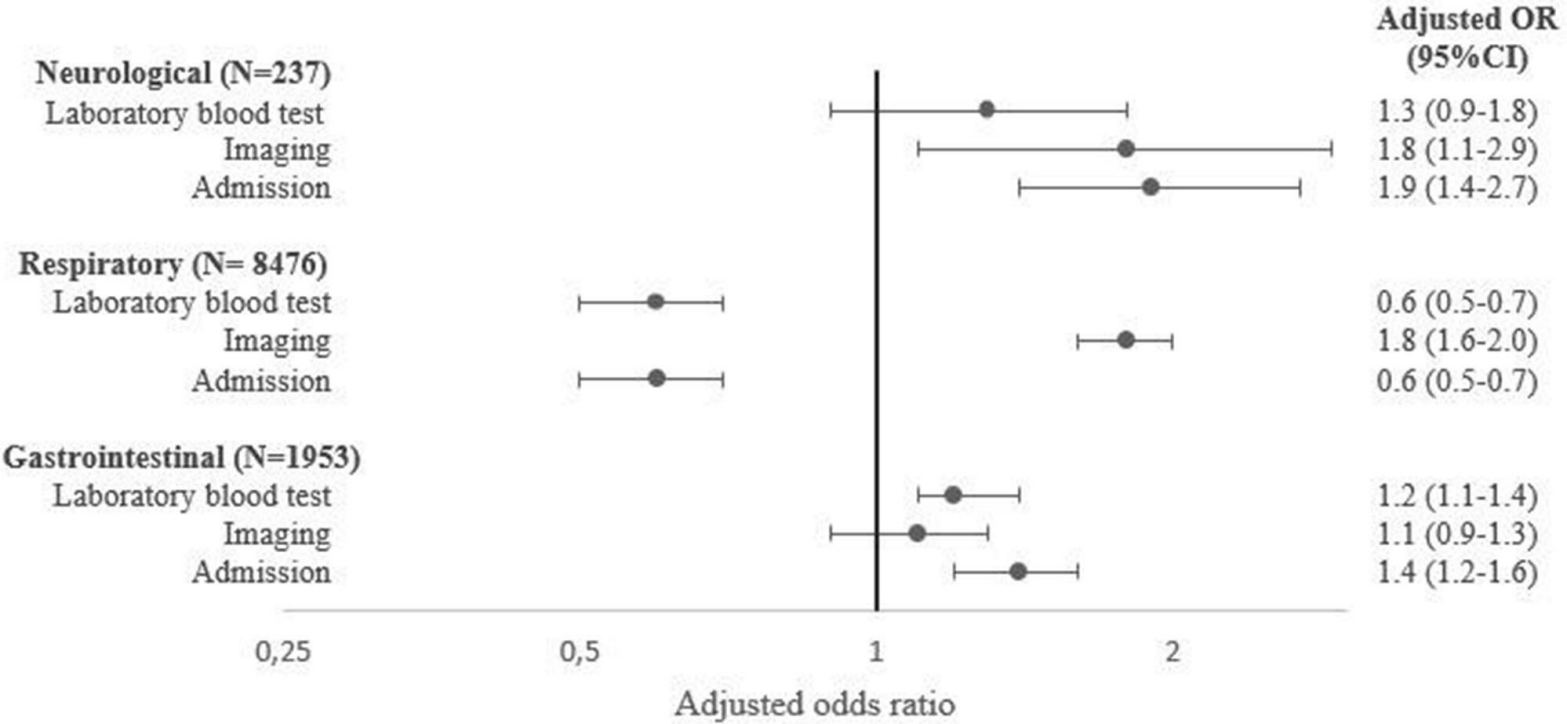
Association between presenting symptoms and management. Other presenting symptoms group as reference group. Adjusted for age, gender, referral status, previous medical care, previous antibiotic use, visiting hours, ED setting.

Management stratified by presenting symptom group is shown in table 3. Imaging was most frequently performed in the respiratory group (15%), while laboratory blood tests were most often performed in the other presenting symptoms groups (41%), and children with neurological symptoms were most often admitted (27%). The association between presenting symptoms and management after adjustment for confounders is shown in the forest plot (figure 1). The neurological group received imaging more often (aOR 1.8, 95% CI 1.1 to 2.9) and were admitted more frequently (aOR 1.9, 95% CI 1.4 to 2.7). The respiratory group had fewer laboratory blood tests performed (aOR 0.6, 95% CI 0.5 to 0.7), were less frequently admitted (aOR 0.6, 95% CI 0.5 to 0.7), but received imaging more often (aOR 1.8, 95% CI 1.6 to 2.0). Lastly, the gastrointestinal group had more laboratory test performed (aOR 1.2. 95% CI 1.1 to 1.4) and were admitted more frequently (aOR 1.4, 95% CI 1.2 to 1.6). Unadjusted ORs are shown in online supplemental appendix E. Stratifying ED settings by low (4 EDs) and high (5 EDs) prevalence of low urgent triaged children showed the same trend, which is shown in online supplemental appendix F.

**Table 3:** Management per presenting symptom group

	Neurological N=237	Respiratory N=8476	Gastrointestinal N=1953	Other N=3473
Laboratory blood test	65 (28)	2473 (29)	777 (40)	1425 (41)
Imaging	24 (10)	1269 (15)	196 (10)	375 (11)
Admission	65 (27)	808 (10)	390 (20)	577 (17)

Absolute numbers and percentages (%) are given.

Additionally, we performed a subgroup analysis stratified for age groups in the respiratory group since they had less laboratory blood testing and were less frequently admitted, and therefore might be suitable for a fast track. Four age groups were created: <2 years, 2<5 years, 5<12 years, 12<18 years. The oldest children had most extensive management with 43% receiving laboratory blood testing, 24% receiving imaging and 15% being admitted (table 4).

**Table 4:** Management in the respiratory subgroup stratified by age groups (n=8476)

	<2 years (n=3263)	2<5 years (n=3130)	5<12 years (n=1648)	12<18 years (n=435)
Laboratory blood testing	878 (27)	923 (30)	485 (29)	187 (43)
Imaging	412 (13)	525 (17)	228 (14)	104 (24)
Admission	369 (11)	266 (9)	106 (6)	67 (15)

Absolute numbers and percentages (%) are given.

## Discussion

More than half (56%) of febrile children attending European EDs are triaged as low urgent, with the majority of this group presenting with respiratory symptoms (60%). Most of the children had the respiratory tract as focus of infection and half of them a presumed viral infection, which is usually self-limiting.^23^ Children with respiratory symptoms had less laboratory blood testing and were less frequently admitted than children in the other presenting symptoms group, although children with respiratory symptoms received more imaging. Most of the imaging performed in this respiratory group were chest X-rays (93%). However, routine chest X-rays are no longer recommended to distinguish between bacterial and viral cases, and treatment decisions are according to the guidelines based on clinical findings.^24^ Moreover, we found that older children with respiratory symptoms had a higher rate of diagnostic tests and 15% required admission. Therefore, we suggest that febrile children with respiratory symptoms are most suitable for a fast track with older children (>12 years) being less suitable since they receive more extensive management than younger children. We deemed children in the neurological group and gastrointestinal group unsuitable for a fast track since they received more laboratory blood testing or imaging and were admitted more frequently compared with the other presenting symptoms group. Previous studies examining the implementation of a fast track at paediatric EDs showed reduced arrival-to-triage times and decreased length of stay of lower acuity patients treated in these units.^5 25^ However, these studies involved broad paediatric ED populations and did not examine subgroups such as children presenting with fever separately.

### Strengths and limitations

The main strength of this study is the use of data from a large cohort of febrile children visiting European EDs increasing generalisability of findings. Additionally, data collection in MOFICHE was extensive, which made it possible to assess management performed in four presenting symptom subgroups to determine which children are most suitable for a fast track. However, several limitations should be mentioned as well. Information on revisits of children was not available in our database. However, revisits do not correspond with inadequate use of a fast track and a previous study showed that low urgent triaged children did not have many revisits with serious illness.^26^ Additionally, our results might not be generalisable to all ED settings, since we included large tertiary hospitals. However, we excluded children with comorbidity in order to make our population more comparable to the paediatric population visiting general hospitals. Furthermore, the large range of 22%–73% of children with low triage urgency attending the participating EDs shows that there is variety in our study population. Finally, our study did not test whether, in practice, these children would have been managed the same way in a fast track or if they would have had shorter stays.

### Implications for clinical practice

Although different streaming approaches might already be in place at ED settings mostly in the UK, this large study across different European EDs show that EDs can direct low urgent triaged febrile children with respiratory symptoms to a fast track based on objective and standardised triage. Implementation of a fast track in emergency care settings might lead to lower medical costs, shorter waiting time and length of stay at the ED for these patients, while improving better patient flow in the rest of the ED.^3 4 10^ For the assessment of children in a fast track, a separate assigned area and the availability of healthcare professionals are required. Having junior doctors or nurse practitioners to clinically assess these children in a fast track and discharge them would be ideal.^27 28^ In general, laboratory blood testing is discouraged in a fast track to ensure a short turnaround time. However, in our study laboratory blood testing in children with respiratory symptoms mostly entailed CRP (99%), which can be performed as point-of-care in a fast track. Most of the European EDs have point-of-care CRP testing available, which can be used in a fast track setting.^29^

Future research is needed in the form of a before/after study or cluster randomised design to compare length of stay, waiting times and revisits before and after implementation of a fast track intervention for low urgent triaged children with respiratory symptoms at paediatric emergency care settings. The effectiveness of implementing a fast track also depends on the patient volume at the ED and the availability of healthcare professionals. Lastly, a fast track should be implemented in routine care as part of the triage process at the ED.

## Conclusion

In this study, we determined that low urgent triaged febrile children with respiratory symptoms were the most suitable for assessment in a fast track. Implementing a fast track for these children presenting to EDs with non-urgent problems could potentially improve patient flow in the ED.

10.1136/emermed-2023-213375.supp2 Abstract translation This web only file has been produced by the BMJ Publishing Group from an electronic file supplied by the author(s) and has not been edited for content.

## Data Availability

Data are available on reasonable request. Data are available from the corresponding author on reasonable request.
