# Supplementary material for: Which low urgent triaged febrile children are suitable for a fast track? An observational European study
Source: Emerg Med J. 2024 Jan 18;41(4):236–41. doi: 10.1136/emermed-2023-213375 (PMC10982627; doi:10.1136/emermed-2023-213375)
Supplement: Abstract translation [file emermed-2023-213375supp002.pdf]

Abstract
Achtergrond

Het aantal kinderen wat de Spoedeisende Hulp (SEH) bezoekt met niet-urgente problemen neemt toe, wat leidt tot een slechte doorstroom van patiënten en drukte op de SEH. Een fast track heeft als doel om de efficiëntie van de evaluatie en het ontslag van laag urgente patiënten te verbeteren. Ons doel was om te identificeren welke kinderen met koorts geschikt zijn voor een fast track gebaseerd op symptomen bij presentatie en het gevoerde beleid.

Methode

Deze studie maakt deel uit van de MOFICHE study, een observationele studie met routinematige data van kinderen met koorts <18 jaar die twaalf Europese SEH's bezoeken. We hebben kinderen met koorts en lage urgentie geïncludeerd (degenen die een triage categorie 'standaard' of 'niet-dringend' hebben gekregen volgens het Manchester Triage System) en hebben kinderen gedefinieerd als geschikt voor fast track wanneer ze minimale diagnostiek hebben ondergaan en naar huis worden ontslagen. De symptomen bij presentatie bestonden uit neurologisch (N=237), respiratoir (N=8476), gastro-intestinaal (N=1953) en andere (N=3473, referentiegroep). Multivariabele logistische regressie analyses met betrekking tot de symptomen bij presentatie en het beleid (bloedonderzoek, beeldvorming en opname) werden verricht met correctie voor de covariaten: patiëntkenmerken, verwijzingsstatus, eerdere medische zorg, eerder antibioticagebruik, bezoekuren en SEH setting.

Resultaten

We includeerden 14.139 kinderen met een gemiddelde leeftijd van 2,7 jaar (IQR 1,3-5,2). Het merendeel had luchtwegklachten (60%), virale infecties (50%) en bestond uit zelfverwijzingen (69%). De neurologische groep kreeg vaker beeldvorming (aOR 1,8, 95%CI 1,1-2,9) en werd vaker opgenomen (aOR 1,9, 95%CI 1,4-2,7). De respiratoire groep kreeg minder bloedonderzoek (aOR 0,6, 95%CI 0,5-0,7), werd minder vaak opgenomen (aOR 0,6, 95%CI 0,5-0,7), maar kreeg vaker beeldvorming (aOR 1,8, 95%CI 1,6-2,0). Verder kreeg de gastro-intestinale groep vaker bloedonderzoek (aOR 1,2, 95%CI 1,1-1,4) en werden ze vaker opgenomen (aOR 1,4, 95%CI 1,2-1,6).

Conclusie

We concludeerden dat laag urgent getrieerde kinderen met koorts en respiratoire symptomen het meest geschikt waren voor een fast track. Deze studie kaart aan welke kinderen getrieerd kunnen worden naar een fast track, waardoor de algehele patiëntenstroom op de SEH mogelijk wordt verbeterd.

This abstract has been translated and adapted from the original English-language content. Translated content is provided on an "as is" basis. Translation accuracy or reliability is not guaranteed or implied. BMJ is not responsible for any errors and omissions arising from translation to the fullest extent permitted by law, BMJ shall not incur any liability, including without limitation, liability for damages, arising from the translated text.
